# Gui Shen Wan ameliorates PCOS-like cellular phenotypes by suppressing TNF-α-mediated inflammation and restoring the PI3K/Akt signaling pathway

**DOI:** 10.3389/fimmu.2026.1739230

**Published:** 2026-03-03

**Authors:** Yan Lu, Lingtong Li, Jia Fang, Wenjuan Ju, Yanfang Yan, Feihua Wu

**Affiliations:** 1 The Affiliated Hospital Of Jiangxi University Of CM, Nanchang, China; 2 The Second School of Clinical Medicine, Guangzhou University of Chinese Medicine, Guangzhou, China

**Keywords:** Gui Shen Wan, network pharmacology, PI3K/Akt signaling pathway, polycystic ovary syndrome, TNF-α

## Abstract

**Background:**

Polycystic ovary syndrome (PCOS) is associated with chronic low-grade inflammation and insulin signaling dysregulation. Gui Shen Wan (GSW), a traditional Chinese medicine formula, has been used empirically for ovarian dysfunction, yet its molecular basis remains incompletely defined. This study aimed to delineate the mechanism by which GSW modulates inflammation-linked insulin signaling in a PCOS-relevant granulosa cell model under metabolic stress, with a focus on the TNF-α/PI3K/Akt axis.

**Methods:**

Network pharmacology based on serum-absorbed constituents identified by UPLC–MS/MS was integrated with *in vitro* validation using dexamethasone- and insulin–challenged human KGN cells to model selected PCOS-relevant cellular phenotypes. The effects of GSW-medicated serum on cell viability, apoptosis, hormone-associated readouts, and inflammatory cytokine production were assessed. PI3K/Akt signaling was examined by Western blotting, and a recombinant TNF-α rescue experiment was performed to probe mechanistic dependence. The effects of candidate constituents (embelin and nobiletin) were further evaluated.

**Results:**

Network pharmacology highlighted TNF and PI3K/Akt signaling as key pathways. In the KGN metabolic stress model, GSW-medicated serum dose-dependently improved cell viability, reduced apoptosis, and attenuated inflammatory cytokine output (TNF-α and IL-6), accompanied by increased phosphorylation of PI3K and Akt. Recombinant TNF-α markedly diminished the protective and signaling-activating effects of GSW, supporting a TNF-α–linked mechanism. Embelin and nobiletin reproduced key anti-inflammatory and signaling effects, and their co-application produced an enhanced combined effect at the tested concentrations.

**Conclusion:**

These findings suggest that GSW mitigates PCOS-like granulosa cell dysfunction under metabolic stress by suppressing TNF-α–associated inflammatory signaling, thereby relieving inhibition of the PI3K/Akt pathway. Given the *in vitro* scope and the medicated-serum approach, the results should be interpreted as mechanistic insight rather than direct evidence of clinical efficacy, and they provide a rationale for subsequent *in vivo* validation.

## 1 Introduction

Polycystic Ovary Syndrome (PCOS) is the most common endocrine and metabolic disorder affecting women of reproductive age, with an estimated global prevalence of 5% to 20% (1, 2). This condition is defined by three cardinal clinical manifestations: hyperandrogenism, persistent anovulation, and the presence of polycystic ovarian morphology. These pathological states are primary contributors to infertility, insulin resistance (IR), obesity, and cardiovascular complications, severely impacting both reproductive health and overall quality of life (3, 4). Critically, PCOS is now recognized as a systemic condition characterized by chronic inflammation, dysregulated steroidogenesis, and autoimmune features, exerting long-term, systemic effects (5). A substantial body of clinical evidence indicates that individuals with PCOS face a significantly elevated long-term risk of endometrial cancer, developing type 2 diabetes, metabolic syndrome, and cardiovascular disease (1, 3). Therefore, elucidating the pathogenesis of PCOS and identifying more effective and safer therapeutic strategies are of paramount scientific and clinical importance for safeguarding women’s health across their lifespan.

The pathophysiology of PCOS is exceedingly complex, wherein chronic low-grade inflammation and insulin resistance (IR) are considered two core, intertwined drivers that create a vicious cycle (6, 7). Studies have demonstrated that levels of various pro-inflammatory cytokines are abnormally elevated in PCOS patients, particularly Tumor Necrosis Factor-alpha (TNF-α), which not only acts as a key mediator of the inflammatory response but also plays a crucial regulatory role within the local ovarian microenvironment (8, 9). Elevated levels of TNF-α are also strongly associated with hyperandrogenism and IR in these patients (10, 11). IR is defined by a diminished biological response of target cells to normal insulin concentrations. The Phosphatidylinositol 3-kinase/Protein Kinase B (PI3K/Akt) signaling pathway is a principal downstream cascade that mediates insulin’s critical functions, including cell proliferation, differentiation, metabolism, and survival (12, 13). A growing body of evidence suggests that pro-inflammatory cytokines such as TNF-α can interfere with the phosphorylation of insulin receptor substrates, thereby inhibiting the proper activation of the PI3K/Akt pathway (14). It should be emphasized that this study focuses on TNF-α-centered inflammatory signaling within granulosa cells rather than a comprehensive immune cell–mediated inflammatory network. While PCOS is a systemic immune-metabolic disorder, granulosa cell–derived cytokines such as TNF-α and IL-6 represent critical local inflammatory drivers within the ovarian microenvironment. Accordingly, the present work is intentionally scoped to a granulosa cell–intrinsic, cytokine-centered mechanism under metabolic stress, rather than systemic endocrine–immune regulation or immune-cell–driven inflammatory networks *in vivo*.

Current clinical management of PCOS is largely symptomatic, relying on treatments such as oral contraceptives to regulate menstrual cycles and reduce androgen levels, or insulin sensitizers like metformin to ameliorate metabolic disturbances. While these approaches can alleviate clinical symptoms to some extent, they often fail to address the fundamental pathology of the disease (15, 16). In this context, Traditional Chinese Medicine (TCM), characterized by its holistic, multi-component, and multi-target approach, is gaining global attention as a complementary and alternative therapy for various complex conditions, ranging from cancer pain management (17) to metabolic and reproductive disorders. Gui Shen Wan (GSW) is a classic formula derived from ancient Chinese medical texts, traditionally used to restore ovarian function (18). Because kidney-deficiency patterns in TCM are often based on chronic inflammation and immune dysregulation as its biological foundation, GSW is hypothesized to possess potential anti-inflammatory properties that warrant investigation. However, its modern pharmacological mechanisms for treating complex diseases like PCOS remain largely uncharacterized and lack high-quality scientific evidence. Notably, other classical formulas for gynecological disorders, such as Guizhi Fuling Wan, have been demonstrated to treat PCOS through mechanisms involving anti-inflammation and improvement of insulin resistance (19–21). This suggests that GSW may possess similar therapeutic potential that warrants in-depth investigation.

Granulosa cell dysfunction represents a key cellular basis of PCOS-associated inflammation and endocrine dysregulation, making it a suitable *in vitro* system to dissect cytokine-driven signaling mechanisms. This study was designed to clarify the potential molecular mechanisms underlying the therapeutic effects of GSW on PCOS. By integrating network pharmacology to identify the common targets between the serum-absorbed components of GSW and PCOS, we predicted key pathways for subsequent *in vitro* validation. Our central hypothesis was that GSW alleviates the inflammatory state in PCOS granulosa cells. We posited this occurs through the downregulation of both the expression and secretion of the pro-inflammatory cytokine TNF-α. This anti-inflammatory effect, subsequently removes the inhibitory pressure on the PI3K/Akt signaling pathway, restoring its proper function and ultimately ameliorating the dysregulation of cell proliferation, apoptosis, and hormone secretion. Furthermore, this study investigates embelin and nobiletin, key active components of GSW, to determine if they exhibit similar therapeutic effects. These findings provide a solid experimental basis for the clinical application of GSW and offer new insights into target development for PCOS therapy.

## 2 Materials and methods

### 2.1 Animals and preparation of medicated serum

All animal experiments were conducted in strict accordance with international guidelines for animal care and use and received approval from the Quanzhou Medical College Experimental Animal Ethics Committee (Approval Number 2024049). This investigation utilized a total of 18 female rats (age: 6 weeks) of the Sprague-Dawley (SD) strain. These animals were supplied by Sipeifu (Beijing) Biotechnology Co., Ltd., which operates under the animal license SCXK(Jing) 2024-0001. Following a 5-day acclimatization period, a randomization process was utilized to allocate the subjects into three distinct experimental arms, with each arm comprising six animals: a control group receiving vehicle, a positive control group treated with 0.2 mg/kg Ethinylestradiol/Cyproterone acetate and 0.23 g/kg Metformin, and the GSW group, which was administered Gui Shen Wan. The formula consists of Cuscutae Semen (Tu Si Zi), Eucommiae Cortex (Du Zhong), Lycii Fructus (Gou Qi Zi), Angelicae Sinensis Radix (Dang Gui), Corni Fructus (Shan Zhu Yu), Rehmanniae Radix Praeparata (Shu Di Huang), Dioscoreae Rhizoma (Shan Yao), and Poria (Fu Ling). The final aqueous extract was concentrated to 1 g crude drug/mL. It was administered at a dosage of 0.1 mL/100g, prepared by the Affiliated Hospital of Jiangxi University of Traditional Chinese Medicine. All treatments were administered once daily via oral gavage for seven days. One hour after the final administration, all rats were anesthetized, and blood samples were collected under sterile conditions from the abdominal aorta for subsequent analysis.

To isolate the serum, collected blood samples were initially allowed to clot at room temperature stand for 3 hours. Following this, the specimens were subjected to a 15-minute centrifugation step, applying a rotational speed of 3,000 rpm. The serum collected from rats within the same experimental group was then pooled and processed. The protocol required a 30-minute incubation at 56 °C within a water bath to achieve heat inactivation. Directly afterward, the serum underwent sterile filtration by passing it through a 0.22 µm micropore filter. The resulting medicated serum was then dispensed into aliquots and stored at -80 °C. Medicated serum from the ethinylestradiol/cyproterone acetate plus metformin group was used as a pharmacological reference reflecting commonly used clinical management for PCOS-associated endocrine and metabolic dysregulation.

### 2.2 UPLC-MS/MS analysis of serum bioactive components

To analyze the bioactive components in the GSW-medicated serum, sample preparation was first conducted. This involved precipitating serum proteins with an acetonitrile/methanol solution, after which the supernatant was collected by centrifugation. This analysis was performed on a UPLC-MS/MS system (ExionLC™ AD, SCIEX). Chromatographic separation was carried out on an Agilent SB-C18 column (1.8 µm, 2.1 mm × 100 mm), utilizing a gradient elution. The mobile phase was composed of 0.1% formic acid in water and acetonitrile. For detection, the mass spectrometry analysis was operated in both positive and negative electrospray ionization (ESI) modes. Metabolite identification was based on fragmentation patterns matched against a self-built database, and quantification was performed in Multiple Reaction Monitoring (MRM) mode by integrating chromatographic peak areas.

### 2.3 Network pharmacology analysis

To elucidate GSW’s underlying mechanisms against PCOS, a strategy based on network pharmacology was employed. Specifically, the bioactive components identified in the GSW-medicated serum via UPLC-MS/MS served as the candidate library for this analysis. Initially, candidate active constituents were screened using pharmacokinetic parameters, specifically oral bioavailability (OB) ≥ 30% and a drug-likeness (DL) score ≥ 0.18. Putative targets for the selected compounds were subsequently predicted via the TCMSP, SwissTargetPrediction, and SymMap platforms. All retrieved target proteins underwent normalization against the UniProt knowledgebase. Concurrently, targets related to PCOS pathogenesis were aggregated from the GeneCards and OMIM resources. GSW’s primary therapeutic targets for PCOS were pinpointed by intersecting the set of drug-related targets with the disease-specific targets. An integrated “drug-component-target” map was thereafter generated and graphically rendered using Cytoscape software (v3.7.2).

For a deeper analysis of the interplay among these core targets, a protein-protein interaction (PPI) network was constructed utilizing information from the STRING database (requiring an interaction score > 0.4). Central (hub) genes within this network were pinpointed via the CytoHubba plugin, integrating the consensus results from five distinct algorithms (MCC, MNC, Degree, EPC, and BottleNeck). Finally, to investigate the pertinent biological functions and signaling cascades, Gene Ontology (GO) and Kyoto Encyclopedia of Genes and Genomes (KEGG) pathway enrichment evaluations were performed. These functional annotations were carried out using the DAVID 6.8 platform, with a P-value < 0.05 set as the criterion for statistical significance. Although these constituents were detected in medicated serum, OB/DL thresholds were applied as a standardized prioritization strategy in network pharmacology; this may bias toward compounds with higher predicted drug-like properties.

### 2.4 Cell culture and PCOS model

The human granulosa cell line KGN was cultured in DMEM/F-12 medium (Gibco, Cat. No. 11320033) supplemented with 10% fetal bovine serum (FBS; Gibco, 10099141) and 1% penicillin-streptomycin (Gibco, 15140122). The cells were maintained in a humidified incubator (Thermo Fisher Scientific, Heracell™ VIOS 160i) at 37°C with an atmosphere of 5% CO_2_. To establish an *in vitro* PCOS model, KGN cells were seeded into culture plates and grown to 70-80% confluency. Subsequently, the culture medium was replaced with a medium containing 1 µmol/L dexamethasone (Sigma-Aldrich, D4902) and 100 nmol/L insulin (Sigma-Aldrich, I9278), and the cells were incubated for an additional 48 hours to induce a PCOS-like cellular phenotype before subsequent treatments. This dexamethasone- and insulin-induced system was used to model key PCOS-relevant cellular features (inflammation, impaired PI3K/Akt activation, apoptosis, and steroidogenic dysregulation) under metabolic stress, rather than to recapitulate the full systemic endocrine–immune phenotype of PCOS. Unlike other ovarian cell lines, KGN cells retain key physiological granulosa cell characteristics, including functional follicle-stimulating hormone receptors and aromatase activity, making them a valid model for investigating steroidogenic and inflammatory responses (22, 23). Model establishment was verified by increased apoptosis and inflammatory cytokine production, together with reduced PI3K/Akt phosphorylation in the Model group, as shown in the Results.

### 2.5 Experimental group design

For the primary efficacy assessment, cells were divided into six groups: a control group treated with blank serum (Control); a model group treated with model serum (Model); a positive control group treated with Ethinylestradiol/Cyproterone acetate + Metformin-medicated serum (Ethinylestradiol/Cyproterone acetate + Metformin); and three experimental groups treated with low (5%), medium (10%), and high (20%) doses of GSW-medicated serum (Low-dose, Mid-dose, and High-dose), respectively. To investigate the causal relationship between inflammation and PI3K/Akt signaling, a mechanism-rescue experiment was performed with the following groups: Control, Model, Model + 20 ng/mL recombinant human TNF-α (TNF-α, PeproTech, 300-01A), Model cells + high-dose GSW serum (GSW), and Model cells + high-dose GSW serum + recombinant human TNF-α (GSW + TNF-α). For the validation of key active components, model cells treated with 15 μM embelin (Low-embelin), 30 μM embelin (High-embelin), 10 μM nobiletin (Low-nobiletin), 20 μM nobiletin (High-nobiletin) or a combination of 15 μM embelin and 10 μM nobiletin (embelin+nobiletin). The concentrations of these monomers were determined based on previous studies (24, 25). Embelin and nobiletin were procured from MedChemExpress under the product codes HY-N0115 and HY-N0145, respectively. For monomer treatments, compounds were dissolved in DMSO, and vehicle controls containing the same final DMSO concentration were included in all experiments.

### 2.6 Cell viability assay

The evaluation of cellular viability was conducted using the Cell Counting Kit-8 (CCK-8) assay (Dojindo, CK04). Initially, KGN cells were dispensed into 96-well plates, with each well containing 3.5 × 10^3^ cells suspended in 100 µL of medium. This was followed by a 24-hour incubation period, allowing cells to adhere and reach approximately 60% confluence. Subsequent to this, the cultures were exposed to differing concentrations of medicated serum for an additional 24-hour duration. Upon completion of the treatment incubation, every well received 10 µL of freshly diluted CCK-8 solution. A final 2-hour incubation of the plates was performed at 37°C, ensuring they were protected from light. Finally, to quantify cell viability, the optical density was recorded at 450 nm utilizing a BIOBASE-EL10A microplate reader.

### 2.7 Apoptosis assay

For every sample, an aliquot of approximately 1 × 10^6^ cells was gathered. The cells underwent two washing cycles with ice-cold PBS (Gibco, 10010023) and were subsequently pelleted via centrifugation (1500 rpm, 3 min). The resulting cellular pellet was resuspended within 300 µL of cold 1× Binding Buffer. A staining solution, consisting of 5 µL of Annexin V-FITC and 10 µL of Propidium Iodide (PI), was then introduced to the cell suspension. After gentle mixing, the mixture was incubated for 10 minutes at ambient temperature while shielded from light. In the final preparation step, an additional 200 µL of cold 1× Binding Buffer was dispensed into each sample. Prompt analysis was then performed by flow cytometry using a FACSCanto II instrument (BD Biosciences).

### 2.8 Enzyme-linked immunosorbent assay

The concentrations of secreted hormones and cytokines in the cell culture supernatant were quantified using commercial ELISA kits. Briefly, following the treatments, supernatants from each group were collected and centrifuged at 1000 × g for 20 minutes to remove cell debris. The levels of Estradiol (RuixinBio, RXJ106056H), LH (RuixinBio, RX106050H), FSH (RuixinBio, RX106046H), Testosterone (RuixinBio, JRX106316), TNF-α (RuixinBio, RX1600891C), and IL-6 (RuixinBio, RXQ96) were then determined. All assays were performed strictly in accordance with the manufacturer’s protocols.

### 2.9 Western blot

To perform immunoblot analysis, total protein was harvested from KGN cells utilizing RIPA lysis buffer (Solarbio, R0020) supplemented with a protease inhibitor (PMSF, Sigma-Aldrich, P7626). Determination of the protein content within the lysates was accomplished via a BCA protein assay kit (Biosharp, BL521A). Identical quantities of protein from every sample were heat-denatured, resolved by 8%-12% SDS-PAGE, and thereafter electro-transferred onto PVDF membranes (Merck Millipore, IPVH00010). Non-specific binding sites on these membranes were blocked at ambient temperature for one hour using 5% non-fat milk dissolved in TBST. Following blocking, the membranes were probed overnight at 4 °C with the following primary antibodies: anti-PI3K (Bioss, BS-10657R, 1:800), anti-p-PI3K (CST, 17366, 1:800), anti-Akt (CST, 9279, 1:800), anti-p-Akt (CST, 4060, 1:1000), anti-PGR (Abcam, AB60954, 1:800), and anti-β-actin (Proteintech, 20536-1-AP, 1:5000). After washing, the membranes were incubated for 1 hour at room temperature with the appropriate HRP-conjugated secondary antibodies (1:10000, Proteintech). Finally, immunoreactive protein bands were visualized with an ECL detection system (Meilunbio, MA0186). Densitometric analysis was conducted using ImageJ, with β-actin serving as the internal loading control. Phosphorylation levels were expressed as the ratio of p-PI3K to total PI3K and p-Akt to total Akt, normalized to β-actin where appropriate.

### 2.10 RT-qPCR

The initial step involved extracting total RNA from KGN cells with the TransZol Up Plus RNA Kit (TransGen Biotech, ER501-01). Following quantification, reverse transcription was conducted using TUAFS SuperMix (TransGen Biotech, AU341); this kit notably includes a procedure for genomic DNA (gDNA) elimination. This newly synthesized cDNA served as the template for quantitative RT-PCR analysis. The reactions were executed on a CFX Connect System (Bio-Rad) utilizing the MagicSYBR Mixture (CWBIO, CW3008). The cycling protocol was programmed for an initial 10-minute denaturation at 95°C, preceding 40 amplification cycles (10 s at 95°C, 30 s at 60°C, and 30 s at 72°C). All oligonucleotide primers, which are detailed in **Table 1**, were commercially produced by Sangon Biotech (Shanghai, China). Calculation of relative mRNA abundance was achieved via the 2^−ΔΔCt^ method, wherein GAPDH served as the endogenous control for normalization.

**Table 1:** Primer sequences used for RT-qPCR.

Gene	Primer	Sequence (5’ to 3’)
TNF-α	Forward	CTCTTCTGCCTGCTGCACTTTG
	Reverse	ATGGGCTACAGGCTTGTCACTC
IL-6	Forward	AGACAGCCACTCACCTCTTCAG
	Reverse	TTCTGCCAGTGCCTCTTTGCTG
GAPDH	Forward	GTCTCCTCTGACTTCAACAGCG
	Reverse	ACCACCCTGTTGCTGTAGCCAA

### 2.11 Statistical analysis

All quantitative data are expressed as the mean ± standard deviation (SD), derived from a minimum of three independent experiments. The statistical analyses were carried out using GraphPad Prism software (version 9.0, GraphPad Software, USA). Prior to analysis, data normality and homogeneity of variance were verified using the Shapiro-Wilk test and Levene’s test, respectively. A one-way Analysis of Variance (ANOVA) followed by Tukey’s *post-hoc* test was applied for comparisons between multiple groups. A p-value less than 0.05 was set as the threshold for statistical significance.

## 3 Results

### 3.1 Network pharmacology analysis predicts key targets of GSW associated with PCOS-relevant pathways

**Figure 1:**
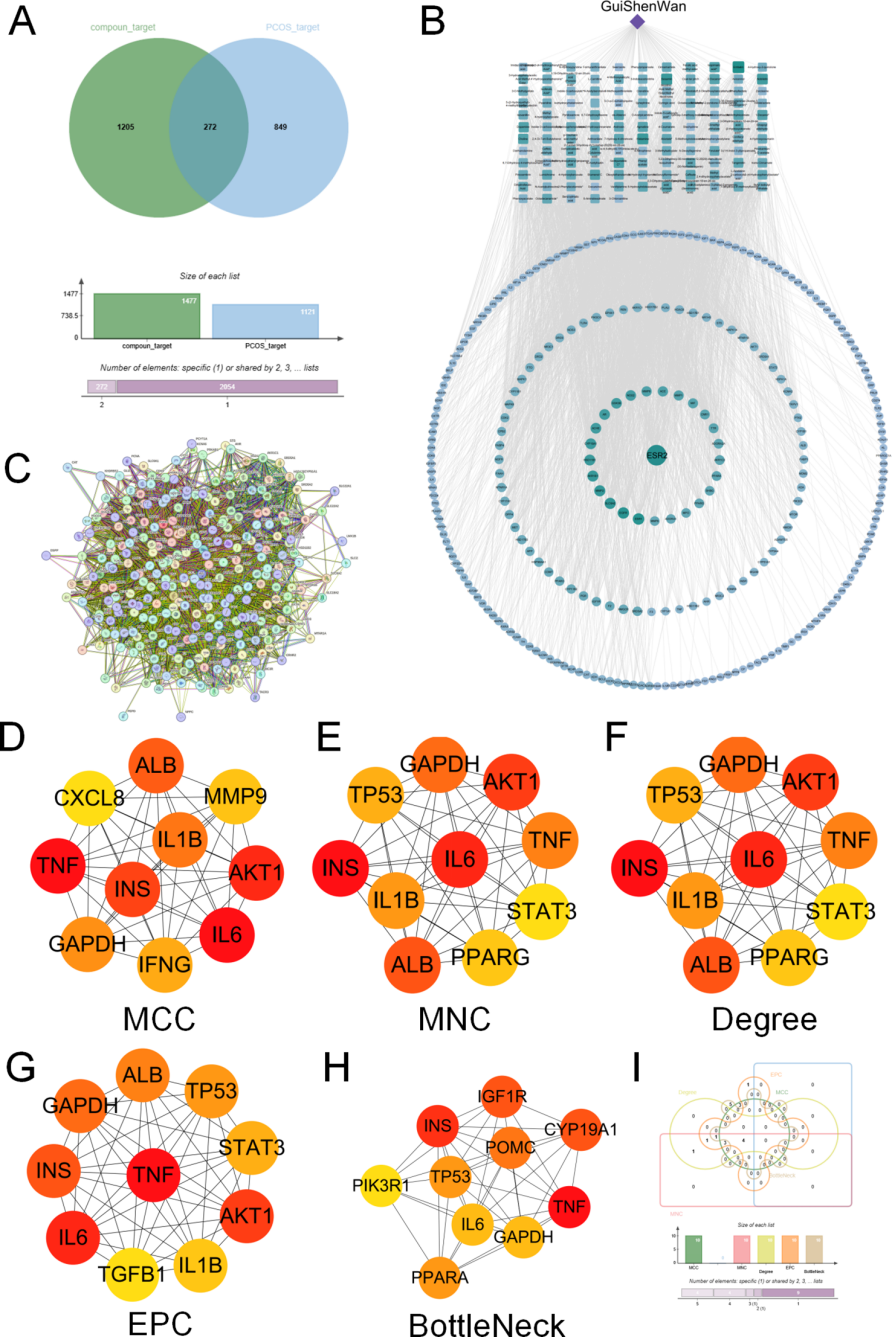
Network pharmacology analysis to identify the key targets of GSW for the treatment of PCOS. **(A)** Venn diagram illustrating overlapping targets between potential targets of GSW’s serum-absorbed components and PCOS-related targets. **(B)** The drug-component-target network of GSW for PCOS. The diamond node represents GSW, rectangular nodes represent active components, and circular nodes represent the common targets. The size and color intensity of the nodes are proportional to their degree, indicating their relative importance in the network. **(C)** PPI network was constructed using the STRING database and visualized in Cytoscape. **(D–H)** Identification of the top hub genes from the PPI network using five distinct algorithms in the CytoHubba plugin: **(D)** MCC, **(E)** MNC, **(F)** Degree, **(G)** EPC, and **(H)** BottleNeck. **(I)** Venn diagram showing the intersection of the hub genes identified by the five algorithms, revealing four core hub genes.

To elucidate the key therapeutic targets of GSW in treating PCOS, we first analyzed its serum-absorbed components (**Supplementary Material S1**). 1477 potential drug targets for these components were predicted using the SwissTargetPrediction, TCMSP, and SymMap databases. This prediction was screened using pharmacokinetic parameters, requiring an OB of ≥ 30% and a DL score ≥ 0.18 (**Figure 1A**).

To illustrate these interactions, a “drug-component-target” map was generated utilizing the Cytoscape software (version 3.7.2). The network included 399 nodes and 3122 edges, with diamond, rectangular, and circular nodes representing the drug, its components, and the targets, respectively. The top five active components, ranked by degree, were embelin, sesamin, nobiletin, dopamine, and histamine. Notably, embelin, sesamin, and nobiletin were associated with the highest number of potential targets, suggesting that these multi-target compounds may play a pivotal role in the therapeutic mechanism of GSW (**Figure 1B**).

To analyze the interplay among these potential targets, a PPI network was constructed for the 272 common targets by querying the STRING database (interaction score > 0.4). This analysis produced a network of 271 nodes and 6863 edges (**Figure 1C**). Hub genes were then obtained from this network by the CytoHubba plugin, which employed five distinct algorithms (MCC, MNC, Degree, EPC, and BottleNeck). A consensus analysis of the leading genes identified by all algorithms yielded four central hub nodes: IL-6, TNF, INS, and GAPDH (**Figures 1D–I**). GAPDH emerged as a hub node likely due to its central role in cellular metabolism and its extensive interactions in protein–protein interaction networks, rather than being interpreted as a specific therapeutic target.

### 3.2 Functional and pathway enrichment analysis of the common targets of GSW and PCOS

**Figure 2:**
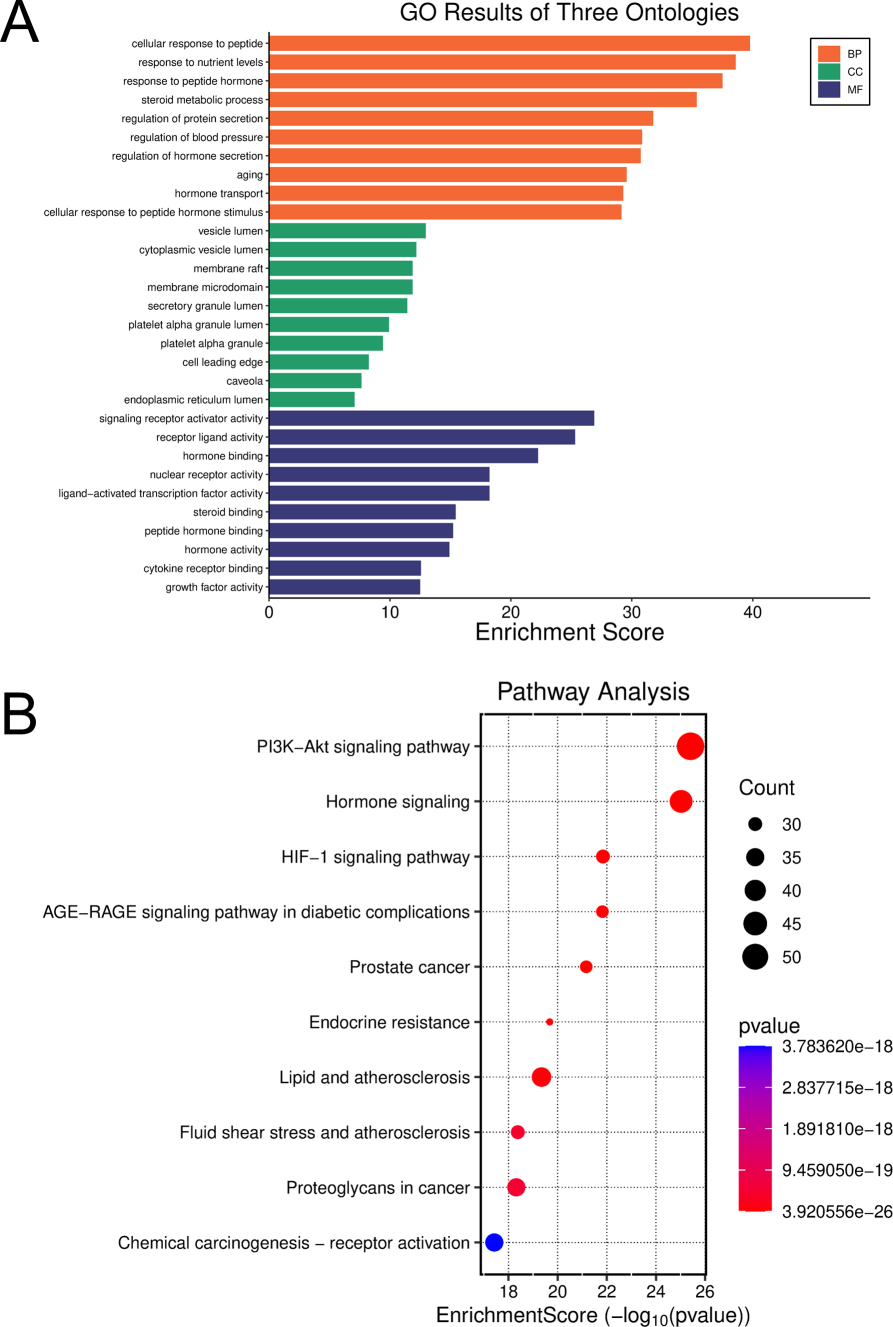
Functional enrichment analysis of the common targets between GSW and PCOS. **(A)** GO enrichment analysis of the common targets. The bar chart displays the top 10 most significantly enriched terms in the categories of BP, CC, and MF. The x-axis represents the gene ratio, which is the proportion of common targets annotated to a specific term. **(B)** KEGG pathway enrichment analysis of the common targets. The bubble plot illustrates the top 10 significantly enriched signaling pathways. The size of each bubble corresponds to the number of genes enriched in that pathway, while the color scale indicates the level of statistical significance (*P*-value). The x-axis represents the gene ratio.

To elucidate the biological roles and molecular cascades that GSW modulates relative to PCOS, GO and KEGG functional enrichment evaluations were conducted on the 272 shared targets, utilizing the DAVID platform. Applying a statistical cutoff of P < 0.05, the Gene Ontology annotation identified 3066 BP items, 113 CC items, and 261 MF items. Within the BP classification, these shared targets showed notable enrichment in functions including the inflammatory response, drug response, response to lipopolysaccharide, steroid metabolism, and the positive control of cell proliferation, suggesting that GSW’s therapeutic effects are closely related to the modulation of inflammation, hormone metabolism, and cell growth status. In the CC category, target proteins were predominantly localized to the extracellular space, plasma membrane, and cytosol, indicating that GSW’s targets are widely distributed, encompassing cell surface receptors, extracellular signaling molecules, and intracellular proteins. In the MF category, the targets were primarily associated with cytokine activity, enzyme binding, and steroid binding, confirming their involvement in complex protein-protein interactions and signal transduction (**Figure 2A**).

The KEGG pathway enrichment evaluation identified 170 pathways showing significant enrichment (P < 0.05), with the leading ten being visualized in a bubble plot. These findings revealed that the common targets were significantly enriched in multiple cascades highly relevant to PCOS pathophysiology. Notably, the Lipid and atherosclerosis pathway was the most highly enriched, consistent with the dyslipidemia commonly associated with PCOS. Furthermore, the IL-17 and TNF signaling pathway were significantly enriched, directly indicating that inflammation is a key aspect of GSW’s mechanism of action. The PI3K-Akt signaling pathway, a critical intracellular hub whose dysfunction is directly linked to insulin resistance in PCOS, was also prominently identified. Other notable enriched pathways included the HIF-1 signaling pathway, Apoptosis, and MAPK signaling pathway (**Figure 2B**).

Collectively, these enrichment analyses suggest that the core mechanism of GSW in treating PCOS involves its broad anti-inflammatory effects and the modulation of key signaling pathways, particularly the PI3K-Akt and TNF pathways, which are closely associated with inflammation, cell metabolism, and survival.

### 3.3 GSW-medicated serum ameliorates proliferation, apoptosis, and hormone secretion in the PCOS cell model

**Figure 3:**
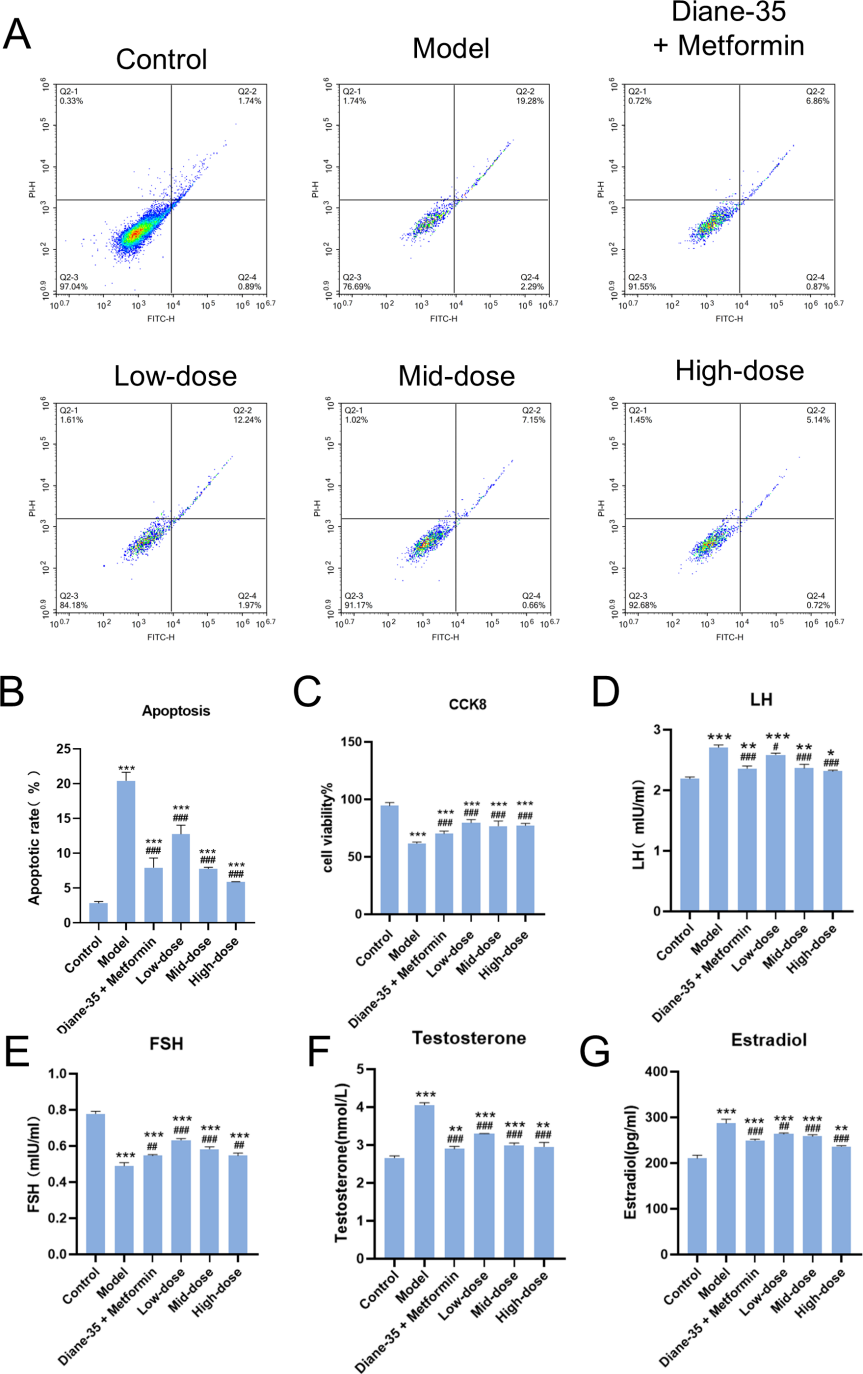
GSW-medicated serum ameliorates apoptosis, proliferation, and hormone secretion in an *in vitro* PCOS cell model. KGN cells were induced into a PCOS-like state and subsequently treated with serum from rats administered a vehicle (Model), a positive control (Ethinylestradiol/Cyproterone acetate + Metformin), or low, medium, and high doses of GSW. **(A)** Representative flow cytometry plots of Annexin V-FITC/PI staining used to assess apoptosis. **(B)** Quantitative analysis of the apoptotic cell percentage across all treatment groups. **(C)** Cell viability determined by the CCK-8 assay after 24 hours of treatment. **(D–G)** Secreted levels of Estradiol, Luteinizing Hormone (LH), Testosterone, and Follicle-Stimulating Hormone (FSH) in the cell culture supernatant, as measured by ELISA. *p < 0.05, **p < 0.01, ***p < 0.001 compared with the control group; ^#^*p* < 0.05, ^##^*p* < 0.01, ^###^*p* < 0.001 compared with the model group. Diane-35 in the figure labels refers to Ethinylestradiol/Cyproterone acetate.

To validate the therapeutic effects of GSW on PCOS, we treated the *in vitro* cell model with GSW-medicated serum. Flow cytometry analysis revealed that the induction of the PCOS model significantly increased the cellular apoptosis rate compared to the control group. Treatment with serum from GSW-administered rats, however, dose-dependently attenuated this pro-apoptotic effect (**Figures 3A, B**). Consistent with these findings, the CCK-8 assay demonstrated that the GSW-medicated serum significantly enhanced the viability of the PCOS model cells in a dose-dependent manner (**Figure 3C**). Furthermore, to elucidate the impact of GSW on hormone secretion, the levels of estradiol, LH, testosterone, and FSH were measured. These data indicated a significant hormonal imbalance in the model group compared to the control. Treatment with GSW-medicated serum led to a dose-dependent decrease in the secretion of estradiol, LH, and testosterone, while concurrently causing a dose-dependent increase in FSH secretion, suggesting that GSW-medicated serum partially restored the steroidogenic/hormone secretion profile in the granulosa cell PCOS-like model (**Figures 3D–G**). Notably, measurements of hormones/cytokines in the culture supernatant reflect *in vitro* outputs under the current experimental conditions and should not be extrapolated to systemic endocrine regulation *in vivo*.

### 3.4 GSW suppresses inflammatory responses and activates the PI3K/Akt signaling pathway in a PCOS cell model

**Figure 4:**
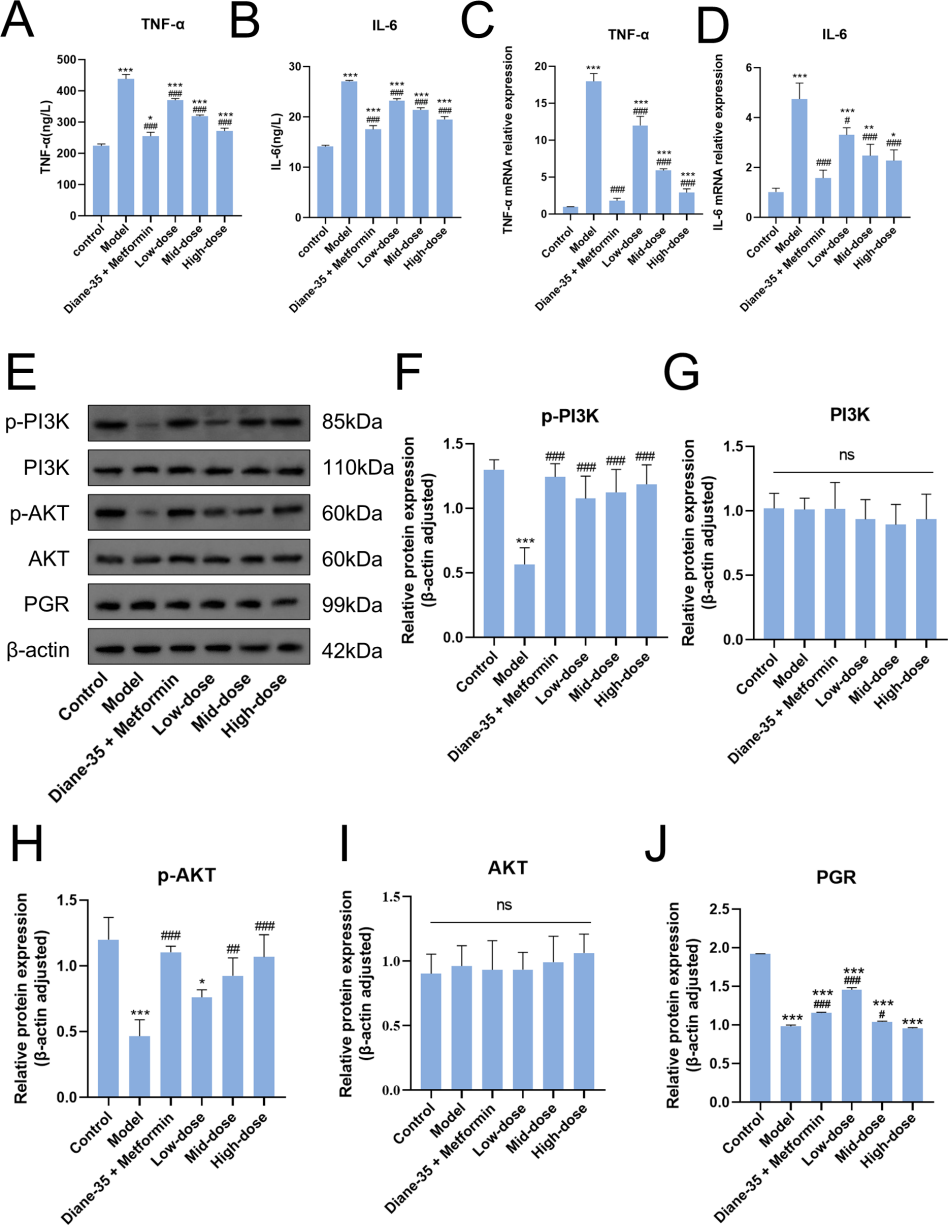
GSW inhibits inflammatory cytokine production and modulates the PI3K/Akt/PGR signaling axis in the PCOS model cells. **(A, B)** Secreted protein levels of IL-6 and TNF-α in the culture supernatant were quantified by ELISA. **(C, D)** Relative mRNA expression levels of IL6 and TNF were determined by RT-qPCR. **(E)** Representative Western blot images showing the protein expression of p-PI3K, PI3K, p-Akt, Akt, and PGR. β-actin was used as an internal loading control. **(F–J)** Densitometric quantification of the relative protein expression levels of p-PI3K, PI3K, p-Akt, Akt, and PGR. *p < 0.05, **p < 0.01, ***p < 0.001 compared with the control group; ^#^*p* < 0.05, ^##^*p* < 0.01, ^###^*p* < 0.001 compared with the model group. Diane-35 in the figure labels refers to Ethinylestradiol/Cyproterone acetate.

Based on the network pharmacology analysis, we investigated the effects of GSW-medicated serum on inflammation and the PI3K/Akt signaling pathway in PCOS model. The results showed a potent anti-inflammatory effect that GSW-medicated serum treatment caused a dose-dependent reduction in both the protein secretion and mRNA expression levels of IL-6 and TNF-α when compared to the model group (**Figures 4A–D**). We then evaluated the impact on the PI3K/Akt signaling cascade. Western blot analysis indicated that GSW-medicated serum, in a dose-dependent manner, significantly increased the phosphorylation levels of both PI3K and Akt. We also assessed the expression of PGR, a critical protein for ovulation. Protein expression of PGR was significantly downregulated in the model group, but GSW-medicated serum treatment effectively restored its levels in a dose-dependent manner (**Figures 4E–J**).

### 3.5 GSW activates the PI3K/Akt pathway by suppressing TNF-α-mediated inhibition

**Figure 5:**
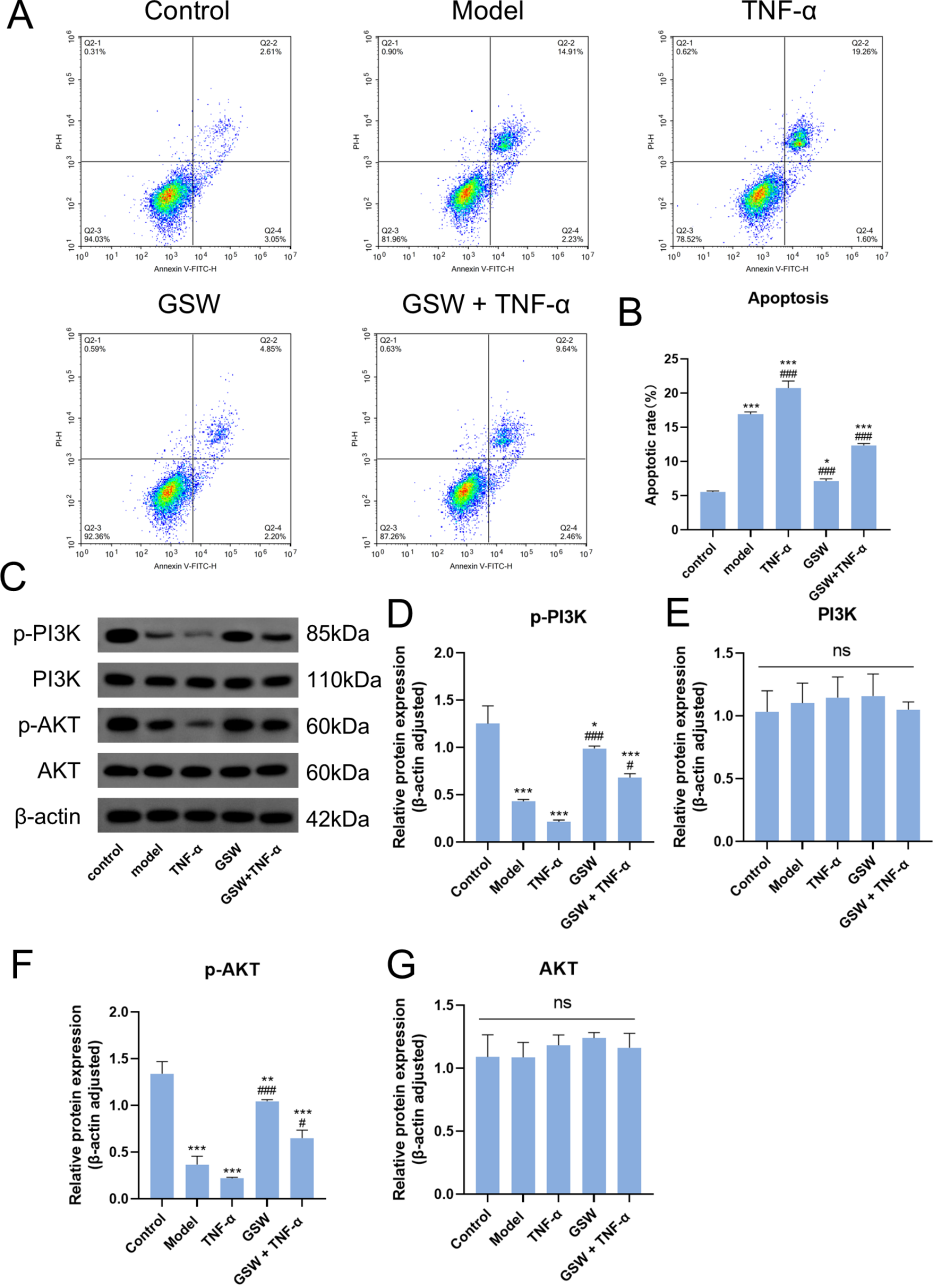
TNF-α attenuates the GSW-mediated suppression of apoptosis and activation of the PI3K/Akt pathway. To elucidate the causal relationship between TNF-α inhibition and PI3K/Akt pathway activation, PCOS model cells were treated with high-dose GSW-medicated serum in the presence or absence of recombinant human TNF-α. **(A)** Representative flow cytometry scatter plots showing apoptosis assessed by Annexin V-FITC/PI staining. **(B)** Quantification of the total apoptotic cell percentage in each treatment group. **(C)** Representative Western blot bands illustrating the protein expression levels of p-PI3K, PI3K, p-Akt, and Akt. β-actin was used as an internal loading control. **(D–G)** Densitometric quantification of the relative protein expression ratios of p-PI3K, PI3K, p-Akt, and Akt. *p < 0.05, **p < 0.01, ***p < 0.001 compared with the control group; ^#^*p* < 0.05, ^##^*p* < 0.01, ^###^*p* < 0.001 compared with the model group.

To further validate the relationship between TNF-α and the PI3K/Akt pathway during GSW treatment, a rescue experiment was conducted using recombinant human TNF-α to interfere with the therapeutic effects of GSW. Flow cytometry analysis revealed that the administration of TNF-α alone exacerbated apoptosis in the PCOS model cells. Critically, when cells were co-treated with GSW and TNF-α, the protective anti-apoptotic effect of GSW was significantly reversed (**Figures 5A, B**).

Western blot analysis further delineated the molecular link between TNF-α and the PI3K/Akt pathway. The phosphorylation levels of PI3K and Akt were significantly downregulated in the model group compared to the control, and this suppression was further exacerbated by the addition of TNF-α. While GSW treatment markedly upregulated the phosphorylation of PI3K and Akt, this activated signaling effect was significantly attenuated by co-incubation with TNF-α (**Figures 5C–G**). The recombinant TNF-α rescue experiment was designed to specifically test the causal relationship between inflammatory suppression and PI3K/Akt activation, rather than to comprehensively map all upstream inflammatory mediators. Taken together, these results strongly suggest that the therapeutic activation of the PI3K/Akt pathway by GSW is dependent on its ability to suppress TNF-α, highlighting TNF-α as a critical therapeutic target of GSW in PCOS.

### 3.6 Embelin and nobiletin as key bioactive components of GSW exerting anti-PCOS effects

**Figure 6:**
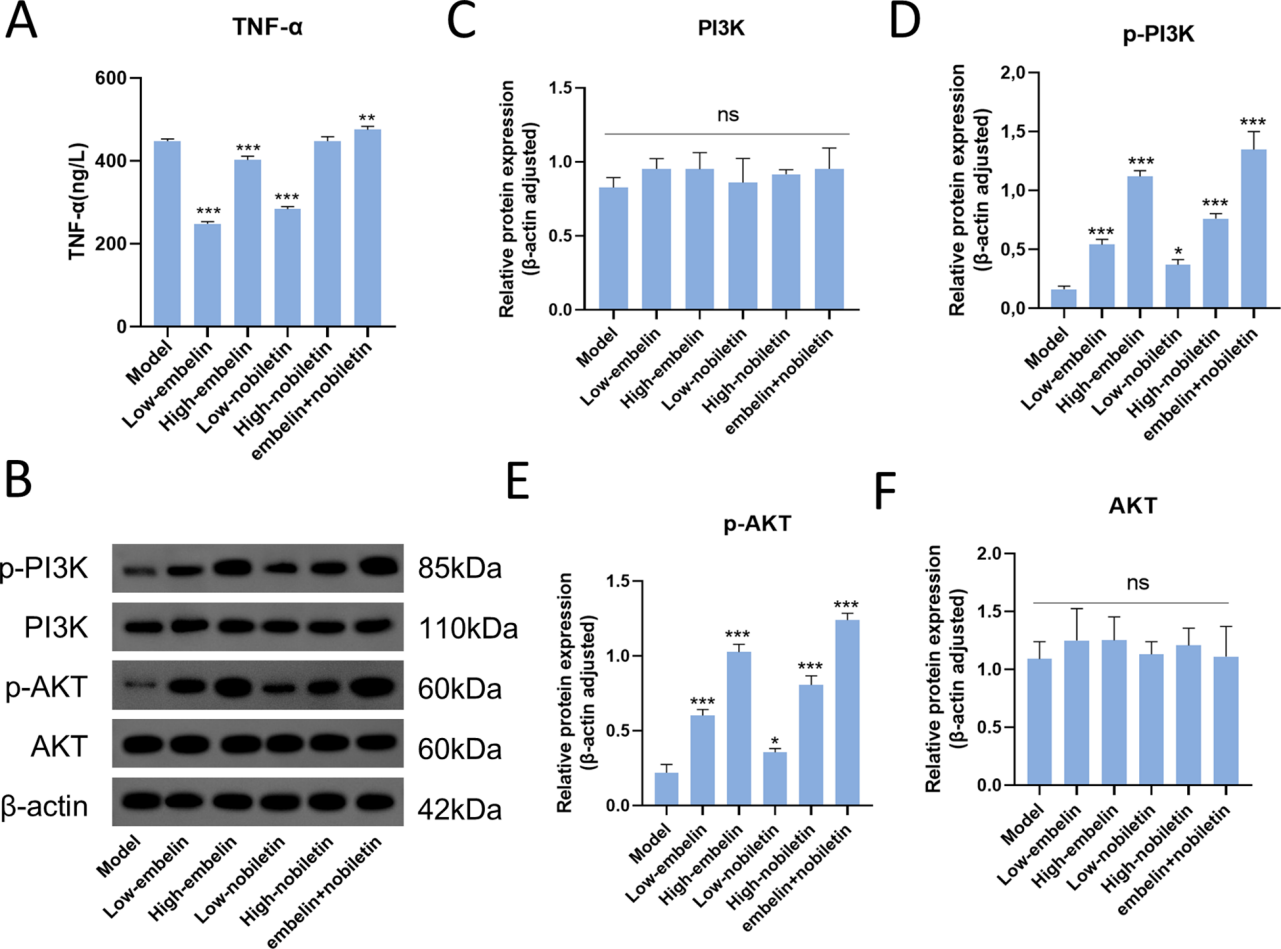
Embelin and nobiletin are key bioactive components of GSW that exert anti-PCOS effects through combined action. To identify the key active constituents of GSW, PCOS model cells were treated with compounds predicted by network pharmacology analysis. **(A)** Screening of the top five predicted compounds (embelin, sesamin, nobiletin, dopamine, and histamine) for their inhibitory effect on TNF-α secretion, as quantified by ELISA. **(B)** Representative Western blot images showing the effects of embelin and nobiletin, at low and high concentrations, alone or in combination, on the phosphorylation levels of PI3K and Akt. β-actin served as a loading control. **(C–F)** Densitometric quantification of the relative protein expression ratios of p-PI3K, PI3K, p-Akt, and Akt. *p < 0.05, **p < 0.01, ***p < 0.001 compared with the control group.

To further identify the key bioactive components of GSW, we treated the PCOS model cells with the top five active ingredients predicted by network pharmacology: embelin, sesamin, nobiletin, dopamine, and histamine. These candidates were selected because they were not only predicted as top-ranking compounds by network pharmacology but were also confirmed to be present in the GSW-medicated serum via UPLC-MS/MS analysis. ELISA results revealed that embelin and nobiletin significantly reduced the secretion of TNF-α, while sesamin showed a modest inhibitory effect. In contrast, dopamine had no significant effect, and histamine slightly increased TNF-α secretion (**Figure 6A**).

Based on these findings, the effects of embelin and nobiletin were investigated further. Western blot analysis demonstrated that both embelin, as well as nobiletin, stimulated the phosphorylation of both PI3K and Akt. This effect was observed in a dose-dependent fashion, as high concentrations yielded a more potent response than low concentrations. Notably, the combined application of low-dose embelin and low-dose nobiletin resulted in a significantly more potent activation of the PI3K/Akt pathway compared to the effect of either compound at low dose level. These results suggest that embelin and nobiletin are key components responsible for the anti-PCOS action of GSW and that they function cooperatively to exert an enhanced effect, consistent with the multi-component therapeutic theory of TCM (**Figures 6B–F**).

## 4 Discussion

This study systematically elucidates the molecular mechanisms by which GSW ameliorates PCOS *in vitro*, by integrating network pharmacology analysis with a series of cellular experiments. Our findings demonstrate that GSW, partly through its active components such as embelin and nobiletin, significantly suppresses TNF-α-mediated inflammatory responses, thereby restoring the impaired PI3K/Akt signaling pathway. This effect enhances proliferation in the PCOS model, suppresses cellular apoptosis, and normalizes the dysregulated production of sex hormones.

A persistent condition of low-grade inflammation represents a critical factor in the pathophysiology of PCOS, contributing to insulin resistance, hyperandrogenism, and ovarian dysfunction (26–28). It is well-documented that individuals with PCOS, encompassing both lean and obese phenotypes, frequently exhibit markedly increased concentrations of pro-inflammatory cytokines such as TNF-α. These cytokines are strongly linked to insulin resistance, hyperandrogenism, and oxidative stress (29–31). Our findings affirm that GSW-medicated serum significantly diminishes not only the protein secretion but also the mRNA expression of pro-inflammatory cytokines, specifically TNF-α and IL-6, in a PCOS cellular model. This indicates that GSW regulates inflammation primarily at the transcriptional level, thereby suppressing the *de novo* synthesis and subsequent release of these cytokines, rather than merely blocking their secretion. Inhibiting these core inflammatory mediators is a crucial strategy for intervening in the progression of PCOS. This finding is consistent with numerous studies on the treatment of PCOS or related inflammatory conditions with TCM. For instance, the YJKL formula was predicted via network pharmacology to target TNF-α in PCOS treatment (32), while the Cuscutae Semen-Salviae Miltiorrhizae formula was shown to ameliorate PCOS by modulating inflammatory factors such as IL-6 and TNF-α (33). Furthermore, TCM formulas like Liu Shen Wan and Jin Gui Shen Qi Wan have been reported to exert anti-inflammatory effects by inhibiting pro-inflammatory cytokines (34, 35). By revealing the anti-inflammatory efficacy of GSW, our study positions it as a potential therapeutic agent for PCOS that acts through an inflammation-centric mechanism, similar to these previously reported TCM formulas. Moreover, therapeutic strategies targeting TNF-α have shown positive outcomes in PCOS animal models; for example, TNF-α inhibitor Etanercept effectively mitigated symptoms and metabolic dysfunction in letrozole-induced PCOS rats, further supporting the importance of anti-inflammatory therapy in PCOS (36).

Our findings emphasize that granulosa cells are active participants in this inflammatory landscape. While conventional views focus on systemic immune cell infiltration, our data suggest that GCs themselves, under metabolic stress induced by insulin and dexamethasone, become a significant source of TNF-α and IL-6. This autonomous cytokine production may contribute to a local inflammatory milieu that impairs PI3K/Akt-mediated insulin signaling and steroidogenesis, thereby linking metabolic stress to reproductive dysfunction.

Our study establishes a link demonstrating that the activation of the PI3K/Akt pathway by GSW is dependent on its inhibition of TNF-α. In the pathogenesis of PCOS, excessive levels of TNF-α are considered a key factor causing insulin resistance and ovarian dysfunction, as it can directly or indirectly suppress the PI3K/Akt signaling pathway (12, 37). The PI3K/Akt pathway is not only implicated in the formation of PCOS but can also be modulated by natural products. For example, Rhei Radix Et Rhizome was found to improve ovarian morphology and hormone levels in PCOS rats by regulating the PI3K/Akt pathway (38). Berberine has also been shown to ameliorate insulin resistance and apoptosis in PCOS rats via the same pathway (39). Furthermore, various interventions, including melatonin and luteolin, have been reported to alleviate PCOS symptoms by activating PI3K/Akt signaling (40, 41). These studies collectively confirmed the crucial role of the PI3K/Akt pathway in the treatment of PCOS. However, GSW is different from single-target drugs or other generic formulations. These findings support a model in which GSW attenuates TNF-α–driven inflammatory signaling while concomitantly restoring PI3K/Akt activity. This TNF-α/PI3K/Akt-centered axis provides a parsimonious mechanistic framework for interpreting the observed cellular phenotypes.

In this study, the key active components embelin and nobiletin were shown to mimic the anti-inflammatory and pathway-activating effects of GSW, with their combined application demonstrating an enhanced effect. Embelin is recognized for its anti-inflammatory capabilities. In parallel, nobiletin, which is a flavonoid, is reported in various studies to possess promising anti-inflammatory, anti-oxidative, and insulin-sensitizing functions. This aligns with findings for other related compounds, such as quercetin. Quercetin is known to suppress inflammation by blocking the TLR/NF-κB pathway, which in turn reduces pro-inflammatory cytokines (TNF-α and IL-6), and simultaneously stimulates the PI3K/Akt signaling cascade (42, 43). The combined action of multi-component, multi-target therapy is a unique advantage of TCM formulas, offering a more comprehensive and durable therapeutic effect. For instance, research on Si Shen Wan has revealed its multi-component enhanced pharmacokinetic effects, indicating that the combined action of multiple active ingredients is necessary for optimal efficacy (44). The combined anti-inflammatory and PI3K/Akt-activating effects of embelin and nobiletin in GSW provide a novel direction for developing natural product-based therapies for PCOS.

Despite the significant progress made, this study has certain limitations. First, the present work is primarily based on an *in vitro* granulosa cell model. While this system allows mechanistic dissection of cytokine-driven signaling events, it does not recapitulate the complex systemic endocrine–metabolic–immune interactions that characterize PCOS *in vivo*. Second, a rat medicated serum approach was employed to reflect *in vivo* absorption and metabolic transformation of the herbal formula. Although this method is widely used in traditional Chinese medicine research, cross-species differences and the heat-inactivation process may influence the stability and composition of bioactive metabolites. Consequently, the observed effects are more likely to reflect conserved inflammatory and metabolic signaling mechanisms rather than species-specific responses. Third, the network pharmacology analysis applied stringent screening criteria (OB ≥ 30% and DL ≥ 0.18), which favor compounds with predicted drug-like properties. This strategy may inadvertently exclude certain bioactive constituents with lower predicted bioavailability that could nonetheless contribute to the therapeutic effects of GSW. Fourth, KGN cells are a tumor-derived granulosa cell line and may not fully mirror the biological behavior of primary human granulosa cells. Therefore, caution is warranted when extrapolating these findings to physiological ovarian function. Fifth, this study focused on TNF-α-centered inflammatory signaling within granulosa cells and did not investigate immune cell–derived inflammatory components, such as macrophage or T-cell involvement, nor broader cytokine networks that may participate in PCOS pathogenesis. Finally, while the key roles of embelin and nobiletin were validated, GSW is a complex formula containing numerous other active components. Whether other constituents also contribute synergistically to the amelioration of PCOS, and the complexity of their interactions warrants further investigation. Moving forward, rigorous clinical trials combined with mechanistic validation, similar to recent comprehensive studies on other TCM formulae (45), are necessary to confirm the therapeutic efficacy of GSW and its active components in PCOS patients.

In conclusion, this study provides evidence that GSW ameliorates the pathological state of PCOS *in vitro* by inhibiting TNF-α-mediated inflammation, thereby restoring the impaired PI3K/Akt signaling pathway. These findings not only provide a solid modern pharmacological basis for the use of GSW in treating PCOS but also highlight the multi-target, holistic advantages of TCM in managing complex diseases. Although these findings provide mechanistic insight into the anti-inflammatory effects of GSW at the cellular level, they should not be interpreted as direct evidence of clinical efficacy. *In vivo* validation and clinical studies are required before translational conclusions can be drawn. This work supports further *in vivo* and clinical investigation of GSW and its active components for PCOS.

## Data Availability

The original contributions presented in the study are included in the article/**Supplementary Material**. Further inquiries can be directed to the corresponding author/s.
